# Assessment of Mosaicism and Detection of Cryptic Alleles in CRISPR/Cas9-Engineered Neurofibromatosis Type 1 and *TP53* Mutant Porcine Models Reveals Overlooked Challenges in Precision Modeling of Human Diseases

**DOI:** 10.3389/fgene.2021.721045

**Published:** 2021-09-23

**Authors:** Clifford Dustin Rubinstein, Dalton T. McLean, Brent P. Lehman, Jennifer J. Meudt, Dominic T. Schomberg, Kathy J. Krentz, Jamie L. Reichert, Mark B. Meyer, Marie Adams, Charles M. Konsitzke, Dhanansayan Shanmuganayagam

**Affiliations:** ^1^Biotechnology Center, University of Wisconsin–Madison, Madison, WI, United States; ^2^Biomedical and Genomic Research Group, Department of Animal and Dairy Sciences, University of Wisconsin–Madison, Madison, WI, United States; ^3^Swine Research and Teaching Center, Department of Animal and Dairy Sciences, University of Wisconsin–Madison, Madison, WI, United States; ^4^Department of Biochemistry, University of Wisconsin–Madison, Madison, WI, United States; ^5^Department of Surgery, University of Wisconsin School of Medicine and Public Health, Madison, WI, United States

**Keywords:** neurofibromatosis type 1, CRISPR, swine, genetic engineering, microinjection, cryptic allele, copy number variation, mosaicism

## Abstract

Genome editing in pigs has been made efficient, practical, and economically viable by the CRISPR/Cas9 platform, representing a promising new era in translational modeling of human disease for research and preclinical development of therapies and devices. Porcine embryo microinjection provides a universally available, efficient option over somatic-cell nuclear transfer, but requires that critical considerations be made in genotypic validation of the models that routinely go unaddressed. Accurate validation of genotypes is especially important when modeling genetic disorders, such as neurofibromatosis type 1 (NF1) that exhibits complex genotype–phenotypic relationships. NF1, an autosomal dominant disorder, is particularly hard to model as it manifests very differently across patients, and even within families, with over 3,000 disease-associated mutations of the neurofibromin 1 (*NF1*) gene identified. The precise nature of the mutations plays a role in the complex phenotypic presentation of the disorder that includes benign and malignant peripheral and central nervous system tumors, a variety of motor deficits and debilitating cognitive impairments and musculoskeletal, cardiovascular, and gastrointestinal disorders. NF1 can also often involve mutations in passenger genes such as *TP53*. In this manuscript, we describe the creation of three novel porcine models of NF1 and a model additionally harboring a mutation in *TP53* by embryo microinjection of CRISPR/Cas9. We present the challenges encountered in validation of genotypes and the methodological strategies developed to counter the hurdles. We present simple options for quantifying level of mosaicism: a quantitative method (targeted amplicon sequencing) for small edits such as SNPs and indels and a semiquantitative method (competitive PCR) for large edits. Characterization of mosaicism allowed for strategic selection of founder pigs for rapid, economical expansion of genetically defined lines. We also present commonly observed unexpected DNA repair products (i.e., structural variants or cryptic alleles) that are refractory to PCR amplification and thus evade detection. We present the use of copy number variance assays to overcome hurdles in detecting cryptic alleles. The report provides a framework for genotypic validation of porcine models created by embryo microinjection and the expansion of lines in an efficient manner.

## Introduction

Pigs provide an ideal translational platform for study of human disease and the development of novel therapies and medical devices due to their similarities to humans in anatomy, physiology, immunology, genetics, and metabolism (Schomberg et al., 2016). Recorded history shows that physicians in ancient Greece (Erasistratus, 304–250BCE) and Rome (Galen, 130–200CE) were using pigs as the earliest homologous models for the study of human biology and disease. Yet, as genetic modeling of human disease increasingly gained importance, transgenic mice became the preferred animal models of choice in research owing to the technical feasibility of genomic manipulations in cultured mouse embryonic stem cells (Yan et al., 2009). Despite the usefulness of mouse models in reductionistic studies, for many diseases, these models have failed to replicate the human pathobiological phenotype due to species-specific differences (Mestas and Hughes, 2004; Lin et al., 2014; Perlman, 2016; Schomberg et al., 2016; Hodge et al., 2019).

In the last decade, the completion of the pig genome sequence (Groenen et al., 2012), the development of high-density SNP chips (Ramos et al., 2009), advances in RNAseq (Isom et al., 2013) and microarray technologies, and the introduction of meganucleases, Zinc Finger Nucleases (Hauschild et al., 2011), Tal effector nucleases (Carlson et al., 2012), and more recently CRISPR/Cas 9 gene editing technologies (Jinek et al., 2012; Cong et al., 2013) have all changed the model creation landscape. The emergence of the genetic information and genome editing technologies, combined with the ability to clone pigs, provided the ability to create novel porcine models (Piedrahita and Olby, 2011; Prather et al., 2013; Yang and Wu, 2018). Genetic disorders that exhibit complex phenotypic presentations are particularly well suited for modeling in pigs and are likely to provide key insights missing from human-rodent comparisons.

Neurofibromatosis type 1 (NF1) exemplifies a phenotypically and genetically complex disorder. NF1 is an autosomal dominant disorder, affecting 1 in 3,000 children worldwide (Gutmann et al., 2017). Individuals with NF1 are prone to the development of benign and malignant peripheral [e.g., neurofibromas, malignant peripheral nerve sheath tumors (MPNST)] and central (e.g., optic pathway glioma, malignant glioma) nervous system tumors. NF1 is also associated with a variety of motor deficits and debilitating cognitive impairments, as well as musculoskeletal, cardiovascular, and gastrointestinal disorders. While mouse models have been valuable in elucidating some of the molecular/cellular pathobiology, congruency in phenotypic presentation of the disorder to those in humans has been poor. Additionally, therapies such as imatinib, shown to be highly effective at attenuating plexiform neurofibroma growth in mouse models of NF1 (Yang et al., 2008), have exhibited far less efficacy for treating human NF1-associated plexiform neurofibromas (Robertson et al., 2012).

The complexity of NF1 is largely due to the variability in mutations that manifest in the large neurofibromin 1 (*NF1*) gene. With over 7,000 human NF1 patients having undergone genetic testing, over 3,000 different germline *NF1* mutations have been identified with little understanding of genotype–phenotype relationship (Gutmann et al., 2017). Neurofibromin 1 is a large and multifunctional protein that is involved in a number of signaling pathways, including the Ras/MAPK pathway, and regulates many fundamental cellular processes (Bergoug et al., 2020). NF1 can manifest because of haploinsufficiency or, in the case of tumors, a biallelic inactivation of the gene (Gutmann et al., 2017). In the latter, other modifying factors including mutations in passenger genes such as *TP53* are frequently involved (Cichowski and Jacks, 2001). Thus, to model NF1 comprehensively, more than one genotypic model is required, and the exact nature of the genotypic modification(s) created in the *NF1* gene and passenger genes needs to be precisely validated.

As biomedical porcine models become increasingly pervasive, the challenge shifts from establishing techniques to create models to establishing methodologies that validate and ensure genotypic precision of these models. Porcine models created by embryo microinjection of CRISPR/Cas9 are particularly susceptible to mosaicism and genotypic variations. While numerous studies have reported the observation of mosaicism in founder models, the focus of those reports has emphasized the generation of the novel model, and less focus has been paid to understanding or overcoming the pitfalls. Finally, due to the long generation time of pigs compared to rodents, it is often more practical to expand novel porcine model lines using presumptive biallelic founders to generate homozygous models in a single generation. However, unexpectedly large rearrangements can evade simple PCR detection methods, and additional validation is required to be assured of the model’s genotype.

Two pig models of NF1, one with a recurrent nonsense mutation and the other with a deletion of exon 42, have been recently developed (Isakson et al., 2018; White et al., 2018). While both models display many of the hallmarks of NF1, given the complexity of the phenotypic profile of the disease and diversity of known genetic variants in human patients, more porcine models of NF1 are needed. Each will have to precisely model the intended mutation with clear validation of genotypic changes created. In this manuscript, we describe the creation of three novel porcine models of NF1 (including a single nucleotide polymorphism, a structural variant, and a splicing mutation) and a model harboring an additional mutation in the passenger gene *TP53*. We detail the challenges encountered in validation of genotypes and the methodological strategies developed to overcome those hurdles and provide a framework for genotypic validation of porcine models created by embryo microinjection.

## Materials and Methods

### CRISPR Design, Synthesis, and Validation

In the conduct of research utilizing recombinant DNA, the investigator adhered to NIH Guidelines for research involving recombinant DNA molecules. Target sites within the genes of interest were selected using Massachusetts Institute of Technology’s CRISPR design tool (now unavailable, previously: crispr.mit.edu,^1^), or later, CRISPOR (Concordet and Haeussler, 2018). All gRNAs were synthesized through *in vitro* transcription (Barnett et al., 2019; Niemi et al., 2019; Wilson et al., 2021). Briefly, we used Phusion polymerase (M0530S; New England Biolabs, Inc., Ipswitch, MA) to synthesize a gRNA *in vitro* transcription template using overlap extension PCR with one primer carrying the T7 sequence, target sequence, and the invariant portion (20bp) of the gRNA sequence for overhang, and another antisense primer carrying the invariant portion of the gRNA sequence (Supplementary Table 1). The *in vitro* template was purified (NucleoSpin Gel & PCR Cleanup, Macherey-Nagel) and used for *in vitro* transcription of gRNA according to the manufacturer instructions (MEGAshortscript T7 Transcription Kit (AM1354); ThermoFisher Scientific, Grand Island, NY). *In vitro* transcription reactions were cleaned up according to manufacturer instructions (MEGAclear Transcription Cleanup Kit (AM1908); ThermoFisher Scientific), with an additional ammonium acetate precipitation and wash with 70% ethanol. Abundance of gRNA was determined by Qubit Fluorometric Quantification (ThermoFisher Scientific).

*In vitro* design validation was performed using a porcine kidney epithelial cell line (LLC-PK1 ATCC CL-101^™^; American Type Culture Collection (ATCC), Manassas, VA) grown in Medium 199 with 3% FBS (Gibco, Thermo Fisher Scientific). Complexes of tracrRNA (Integrated DNA Technologies (IDT), Coralville, IA) and target-specific crRNA (IDT) were generated by heating 1:1 crRNA/tracrRNA by mole to 95°C and cooled to 25°C at 0.1°C/s. A total of 0.2×10^6^ cells were mixed with a final concentration of 2μm crRNA/tracrRNA, 1.95μm Cas9 (Cas9 Nuclease V3; IDT), 2μm electroporation enhancer (IDT), and 1μm single-stranded DNA donor (Ultramer; IDT) using the SF Cell Line 4D-Nucleofector^™^ X Kit S (Lonza, Basel, Switzerland) and the EN-150 protocol of a 4D Nucleofector^™^ (Lonza). Subsequently, cells were recovered in complete growth media for 48h, lysed, and the targeted region was PCR amplified with the hotstart PrimeSTAR GXL DNA polymerase (Takara Bio Inc., Mountain View, CA) and sequenced using the MiSeq platform (Illumina Inc., San Diego, CA; see “Genotyping” subsection below).

### Estrus Synchronization, Superovulation, Artificial Insemination, and Embryo Retrieval From Donor Pigs

Experiments involving animals were conducted under protocols approved by the University of Wisconsin–Madison Institutional Animal Care and Use Committee in accordance with published National Institutes of Health and United States Department of Agriculture guidelines. The segments of the methodology utilizing animals were conducted at the UW Swine Research and Teaching Center (SRTC), a specific pathogen-free (SPF) breeding, housing and surgical facility with the capacity to house up to 1,600 pigs, and the capability to raise piglets to maturity.

**Figure 1:**
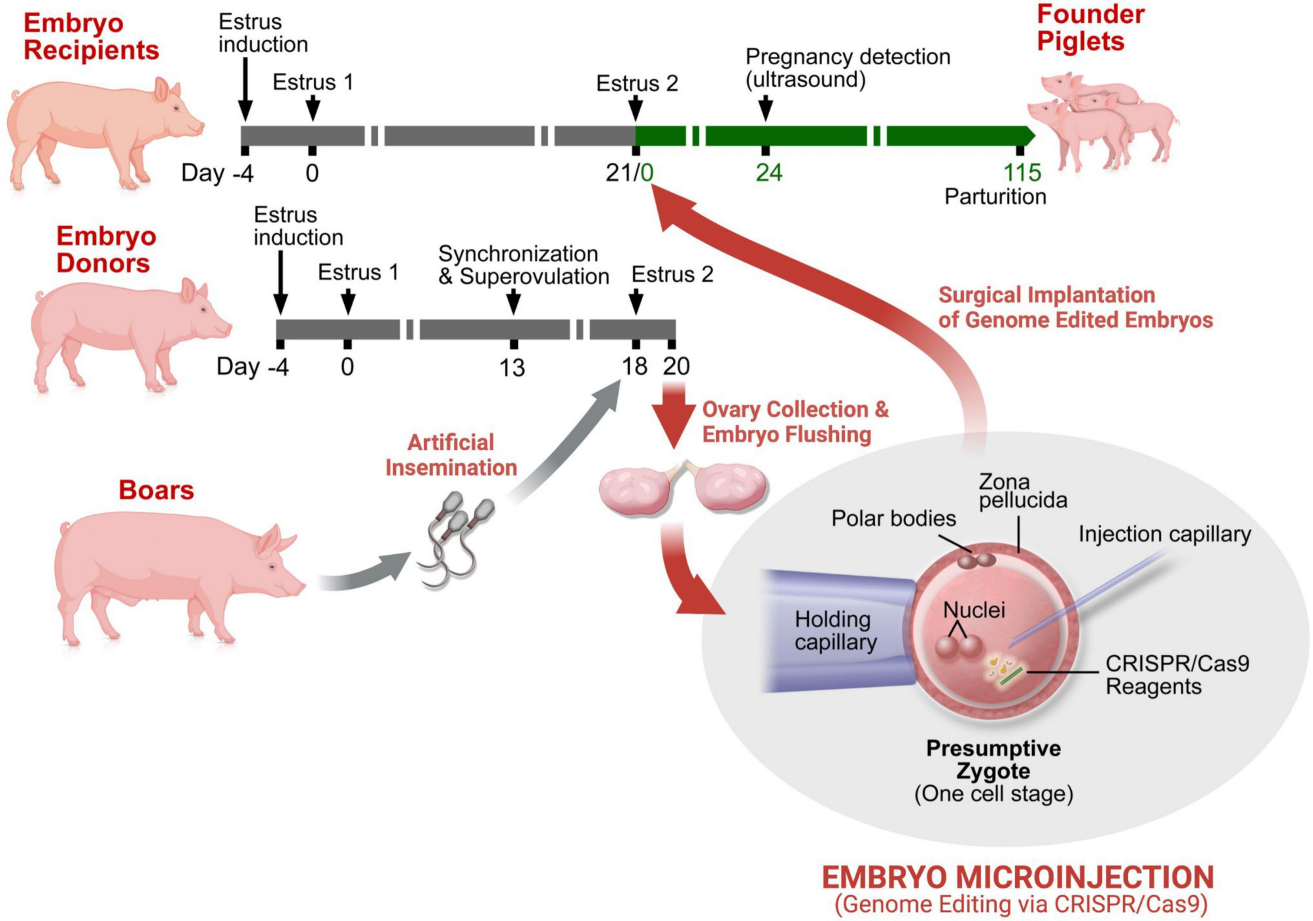
Genetic engineering of pigs by embryo microinjection. Estrus synchronization of potential embryo recipient (surrogate pigs) and donor pigs is achieved by a refined hormone regimen. A variation in the regimen is utilized in the embryo donors to increase ovulation rate and the potential for higher yield of one-cell embryos (zygotes). The embryo donors are artificially inseminated on second estrus detection and euthanized shortly thereafter for collection of single-cell presumptive zygotes. The isolated zygotes are microinjected with the CRISPR/Cas9 components and implanted into the oviducts of surrogate pigs by surgical access usually within 4–6h of initial embryo collection. Transabdominal ultrasound is used to confirm pregnancy and to track fetal development. Shortly after parturition, genomic DNA is obtained from the neonatal piglets for genotypic validation and identification of genetically engineered “founders” for subsequent breeding and expansion of the novel swine line.

Our process for creating genetically engineered NF1 porcine models is summarized in Figure 1. Initial estrus was induced (Day -4) in female pigs by intramuscular (i.m.) administration of 5ml of P.G. 600® (Intervet Inc (Merck Animal Health), Madison, NJ); a mixture of pregnant mare serum gonadotropin and human chorionic gonadotropin (hCG)). Estrus detection (once daily), using “detection of standing heat” methodology commonly used in swine breeding (Worwood, 2007), was started three days after induction. Pigs exhibiting synchronized estrus were designated as “embryo donors” (Day 0). Thirteen days following estrus detection, the follicular phase of the donors was synchronized by i.m. administration of 20mg prostaglandin F_2α_ (Lutalyse^®^; Zoetis Inc., Kalamazoo, MI). Prostaglandin F_2α_ regresses the corpora lutea responsible for the maintenance of the luteal phase, thereby synchronizing the follicular phase of estrus. The use of Lutalyse^®^ significantly increases ovulation rate and one-cell embryo recovery (Sommer et al., 2011). Sixteen hours after prostaglandin F_2α_ administration, superovulation was induced by administration of prostaglandin F_2α_ (20mg, i.m.) and P.G. 600^®^ (7.5ml, i.m.) and then hCG [1,000IU, subcutaneous; 72h after Lutalyse and P.G. 600^®^ administration; Chorulon, Intervet Inc (Merck Animal Health, Summit, NJ)]. Second estrus detection (twice daily) and artificial insemination (twice daily) occurred on Days 18 and 19. On Day 20, the embryo donors were euthanized and their reproductive tracts were exposed *via* abdominal incision and oviducts ligated at the uterotubal junction. Each ovary and oviduct were aseptically dissected out and transported in a portable incubator to the laboratory where oviducts were dissected from the ovaries. Oviducts were flushed with phosphate-buffered saline supplemented with 1% newborn calf serum (1% NBCS PBS). Single-cell presumptive zygotes were identified under a dissecting microscope and moved into pre-equilibrated modified porcine zygote medium (PZM3-MUI; Whitworth et al., 2014).

### Embryo Microinjection of CRISPR/Cas9

Up to 50 zygotes were moved into HEPES-buffered media for cytoplasmic microinjection of the CRISPR/Cas9 editing components. Each zygote was held in place with a holding pipette while the microinjection pipette delivered reagents into the cytoplasm. The injection solution contained gRNAs, *S. pyogenes* Cas9 mRNA (PNA Bio Inc., Thousand Oaks, CA or Millipore Sigma, Burlington, MA), and single-stranded donors (when necessary) to create the *NF1* or *TP53* mutations. The microinjected presumptive zygotes were maintained at 39°C in a 5% CO_2_ and 95% air mixture, when not being manipulated. The microinjections were performed by the Animal Models core within the University of Wisconsin-Madison Biotechnology Center.

### Estrus Synchronization, Embryo Transfer, Pregnancy, and Parturition in Surrogate Pigs

Initial estrus was induced (Day -6) in female pigs by intramuscular (i.m.) administration of 5ml of P.G. 600^®^. Estrus detection (once daily) was performed and animals showing signs of estrus were retained as potential surrogates (“embryo recipients”). Heat detection was performed twice daily for the second cycle, and pigs displaying estrus 18 to 24h after embryo donors were designated as the ideal surrogates. Within hours of microinjection, presumptive zygotes were implanted into the oviduct of the surrogates *via* laparotomy. Briefly, under surgical anesthesia, the reproductive tracts of the surrogates were accessed *via* a midline abdominal incision. Up to 150 presumptive zygotes were transferred into each surrogate in one oviduct, minimizing culture media volume. On recovery, appropriate post-operative [antibiotic and non-steroidal anti-inflammatory (NSAID)] care was provided. Transabdominal ultrasound was used to confirm pregnancy and to track fetal development as appropriate. Pregnant surrogates were transferred into farrowing pens one week prior to expected parturition (115–117days after estrus). Four days after parturition, a non-steroidal anti-inflammatory (NSAID) drug was administered to the neonatal piglets and tail biopsies were obtained for genotypic validation.

### Genotyping

Genomic DNA from tail biopsies of newborn piglets was extracted using an overnight 55°C incubation in genomic lysis buffer (20mm Tris–HCl, pH8; 150mm NaCl; 100mm EDTA, 1% SDS) with 100μg/ml proteinase K (Promega, Madison, WI) digestion, followed by protein precipitation (Protein Precipitation solution, Promega). The supernatant was then precipitated in isopropanol; the pellet was washed with 70% ethanol and resuspended in sterile ddH_2_O. PCR was performed using Q5® Hot Start High-Fidelity DNA Polymerase (New England Biolabs, Inc.) according to manufacturer’s suggestions (Supplementary Table 2). Sanger sequencing was performed on PCR amplicons using primers that generated the amplicons on a 3730xl Genetic Analyzer (Thermo Fisher Scientific). Targeted amplicon sequencing (TAm-Seq) was performed by amplifying targeted genomic regions with primers carrying indexing adapters, followed by 0.7X bead purification (Axygen AxyPrep Magnetic Bead Purification Kit, Corning). An 8-cycle indexing reaction was performed using custom combinatorial dual indexing primers, followed by another 0.7X bead purification and sample pooling. TAm-Seq of pooled samples was performed on a MiSeq v2 Nano 2×250 flow cell (Illumina). Sequencing data were demultiplexed and analyzed using CRISPResso or CRISPResso2 (Pinello et al., 2016; Clement et al., 2019). Read depths of at least 2,000X were acquired, providing sensitivity to reliably detect rare alleles at <0.1% abundance (Hendel et al., 2015). Sanger sequencing was used to characterize breakpoints of excisions, while TAm-Seq was used to detect indels and SNPs. The sequencing was performed by the University of Wisconsin-Madison DNA Sequencing Facility.

### Statistical Analysis

Logistic regressions were performed in JMP (JMP Pro 15.0.0, SAS Institute Inc., Cary, NC) using the presence or absence of germline transmission in progeny as the dependent categorical response and allelic abundance of the edited allele (pixel density or Illumina read representation) as the independent continuous regressor.

### Droplet Digital PCR

Copy number variation (CNV) analysis was performed using digital PCR (dPCR; QX200 Droplet Digital PCR System, Bio-Rad Laboratories, Inc.). Purified genomic DNA was digested using BamH1 (New England Biolabs, Inc.) and then further diluted 1:10 using sterile ddH_2_O. dPCR reactions were performed according to the manufacturer’s suggestions such that the final concentrations of primers and probes were 900nM and 250nM, respectively. PCR conditions were used according to manufacturer recommendations, except for a 50°C annealing temperature for 10s (Supplementary Table 2). Droplets were analyzed on a QX200 droplet reader (Bio-Rad), and CNV was calculated using RPP30 as a reference gene.

## Results and Discussion

**Figure 2:**
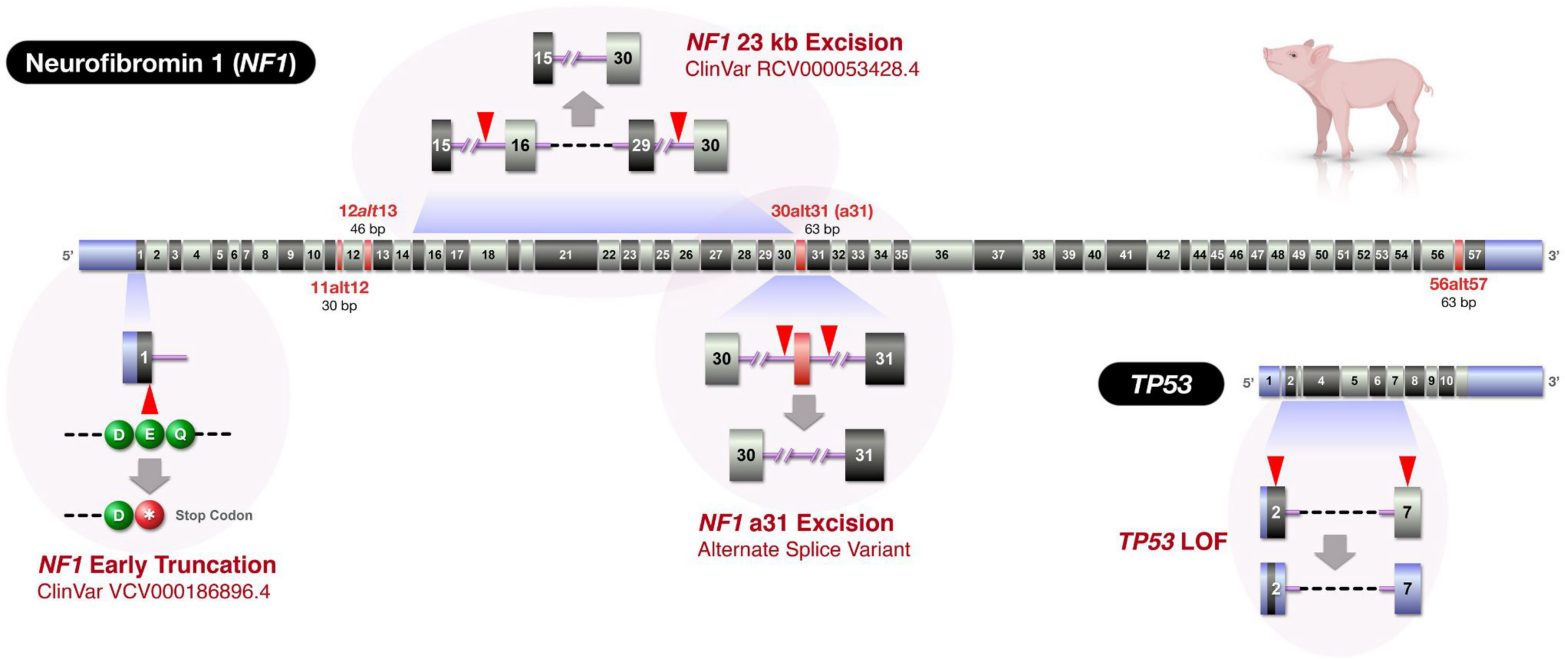
Swine *NF1* and *TP53* gene maps and locations of genetic alterations in the novel swine models. The figure depicts three *NF1* edits and a *TP53* edit. Due to the large size of the *NF1* gene, the figure only displays exonic regions (to relative scale) of the two genes (*NF1*: ENSSSCT00000019317.4 and *TP53*: ENSSSCT00000019534.4). Exons displayed as red indicate alternatively spliced exons homologous to those in humans. The exon numbering was based on Ensembl Sscrofa11.1, but alternatively spliced exons were added to provide congruence with previously published human NF1 nomenclature (Anastasaki et al., 2017). When intron lengths cannot be depicted to scale, double slashes are displayed. Blue exonic regions: untranslated regions. Red arrowheads: Cas9 target sites.

The creation of genetic porcine models is primarily achieved *via* two distinct pipelines: by somatic cell nuclear transfer (SCNT) or by embryo microinjection (Yang and Wu, 2018). The use of SCNT to reproductively clone a new line of pigs using genetic-edited nuclear donor cells allows for more complex genetic manipulations or multiple edits to be achieved with *in vitro* genotypic confirmation prior to creating live founder pigs. However, porcine SCNT has a higher barrier to entry and requires dedicated, costly equipment, and unique expertise. The efficiency of cloning pigs by SCNT is very low and can be as low as 0.2% in some pig breeds (Zhao et al., 2009). Thus, the process requires a large number of ova for embryo reconstruction to produce a few cloned piglets. Most research groups thus rely on ova obtained from large swine abattoirs to make the process practical. This introduces a significant biosecurity risk that many research swine facilities are not willing to take. In contrast, embryo microinjection requires a more universally available set of equipment and skills and has improved embryo viability over SCNT. Microinjection works well for generating genome-edits that occur relatively efficiently (e.g., knockouts, excisions, and single nucleotide polymorphisms), but is a less reliable strategy for more difficult edits (large knock-ins, e.g., fluorescent tagging, domain swapping; Peng et al., 2015). Microinjection-based engineering also introduces other challenges including mosaicism (Mehravar et al., 2019) and the inability to prescreen nuclei against unwanted large genomic rearrangements and structural variants (Shin et al., 2017). Despite these challenges, microinjection remains a viable strategy for creating novel genetic porcine models. While we can use either of the pipelines, for logistic reasons, we relied on embryo microinjection approach to create our novel porcine models of NF1 targeting three different regions of the *NF1* gene and a region of the *TP53* gene (Figure 2).

### *NF1* Early Truncation (Pathogenic Variant) Model

**Figure 3:**
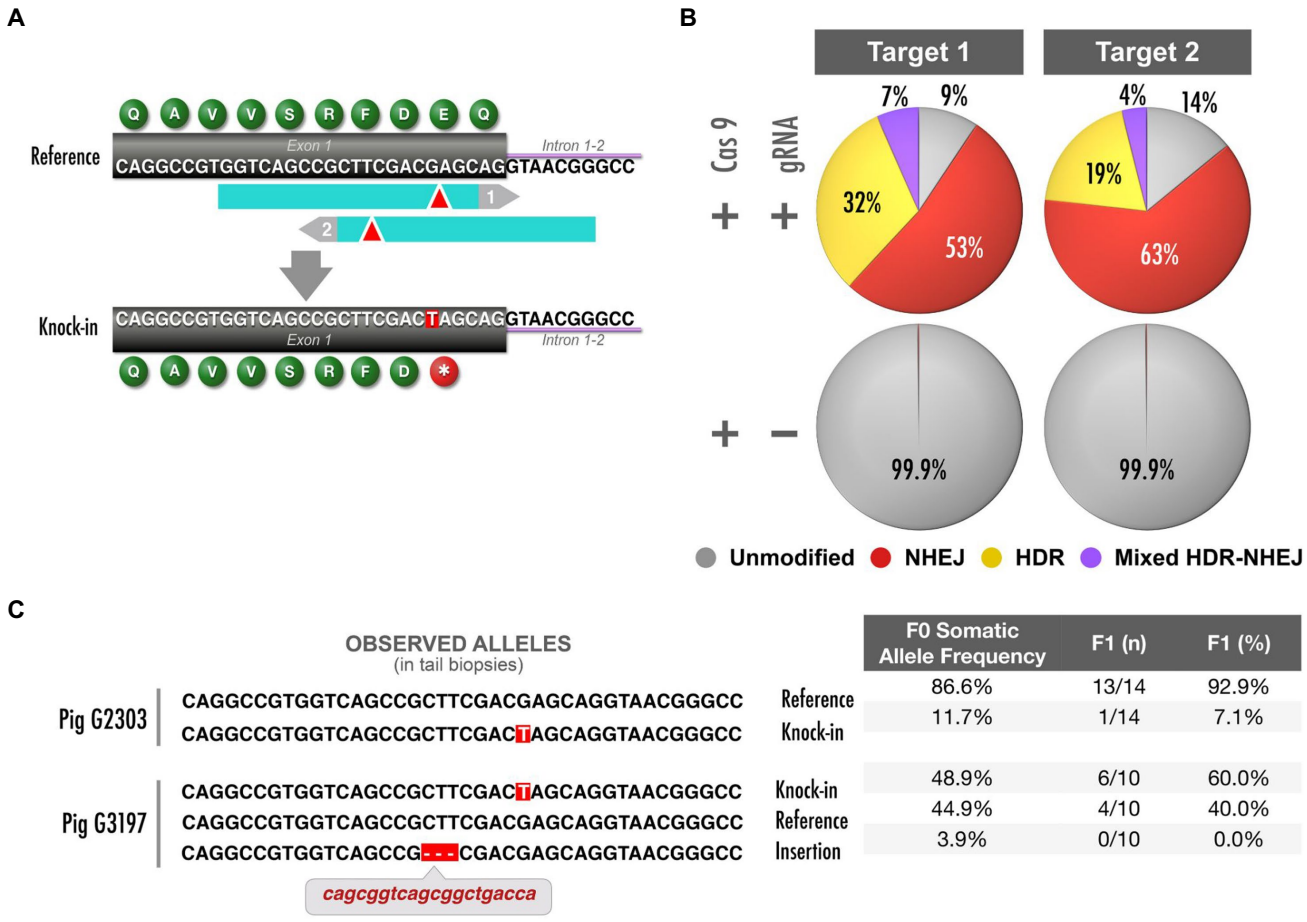
*NF1* early truncation model and analysis of mosaicism. (**A**) Top: schematic depicts *NF1* exon 1 and two Cas9 target sites (blue, PAM in gray); donor oligonucleotides are not shown (Supplementary Table 3). Bottom: precise G>T mutation generates an early truncation codon. (**B**) *In vitro* validation of targets shown in (A). Quantification of DNA repair products through TAm-Seq of amplicons and CRISPResso analysis. (**C**) Of four founders in total, two representative, validated founders were bred to generate a herd of E19^*^ F1 pigs. Left: allele sequences detected in somatic tail tissue from these founders (>1%). Right: Quantification of abundance of each allele and the proportion of sired F1 pigs that carry each allele from each founder. The pig with higher abundance of E19^*^ in somatic tissue yielded a higher frequency of E19^*^ offspring (logistic regression, *p*<0.005).

One of the models that we pursued introduced a human variant designated as pathogenic in ClinVar (dbSNP: rs786203307; ClinVar VCV000186896.4) and has been identified in patients from several studies (Fahsold et al., 2000; Valero et al., 2011; Hutter et al., 2016). This mutation consists of a G to T transversion located in coding exon 1 of *NF1*, resulting in early truncation at the 19th amino acid of neurofibromin 1 (ENSSSCT00000019317.4; p.Glu19Ter, or abbreviated as E19^*^; Figure 3A).

First, we used an *in vitro* validation assay utilizing LLC-PK1 cells to design and select the optimal genome editing reagents. We tested two gRNA–donor pairs (Supplementary Table 3) and quantified the frequency of alleles repaired through HDR to generate E19^*^. Both CRISPR targets were relatively comparable in their HDR efficiency (Figure 3B). Furthermore, the potential off-target profile was highly specific and similar for both pairs (Concordet and Haeussler, 2018): Targets 1 and 2 did not have off-target sequences that would be predicted to be edited (neither target had off-targets with two or fewer mismatches nor off-target CFD scores >0.5; Doench et al., 2016). Expanding the analysis to consider off-target regions that are unlikely to be edited, Targets 1 and 2 only had four and two potential off-targets, respectively, with three mismatches. However, an algorithm trained on vertebrate oocytes (Moreno-Mateos et al., 2015) predicted the editing efficiency to be higher at target 2. Thus, this design was selected to advance to embryo microinjection. We recognize that this decision tree should not be a universal workflow for all edits, and that each edit’s unique circumstances should be considered. After two rounds of embryo microinjection and surgical implantation into recipient sows (Figure 1), we produced 38 live piglets. After delivery of resultant piglets, tail samples were taken for molecular characterization. The targeted region of exon 1 was PCR amplified for TAm-Seq, yielding the identification of four animals carrying the E19^*^ knock-in allele (Figure 3C).

Although embryos were injected at the single-cell stage, Cas9 activity and DNA repair may have occurred after the initiation of embryonic cleavage and initiation of mitotic divisions, such that different cells carry distinct DNA repair outcomes (mosaicism; Mehravar et al., 2019). Primordial germ cells are not committed to the germline fate until gastrulation and epiblast formation (Lawson and Hage, 1994; Tanaka and Matsui, 2002); therefore, we presume the germline and somatic layers are composed of roughly equivalent populations of mosaic cells (Oliver et al., 2015); our extensive data (not shown) from creation of mouse models also support this assumption. Consequently, the abundance of desired alleles in somatic tail tissue would reflect the abundance of desired alleles in the germline. Since TAm-Seq provides a reliable measurement of the relative abundance of desired alleles, we used the abundance of E19^*^ within the Illumina library generated from tail biopsies as a measurement of the pervasiveness of the E19^*^ allele within the mosaic embryo, and thus its germline.

Using the generated data, we selected and bred the animals with a range of abundance of the E19^*^ allele (Figure 3C). Indeed, the founder pig (G3197) that had higher abundance of the E19^*^ allele in somatic tissue resulted in a higher rate of germline transmitted F1 piglets (logistic regression, *p*<0.005). Estimating allele frequency in somatic tissue allowed us to reliably predict the frequency of germline transmission. Thus, with this method, mosaic founders can be used to reliably and efficiently generate a large herd.

### *NF1* 23Kb Excision (Pathogenic Variant) Model

**Figure 4:**
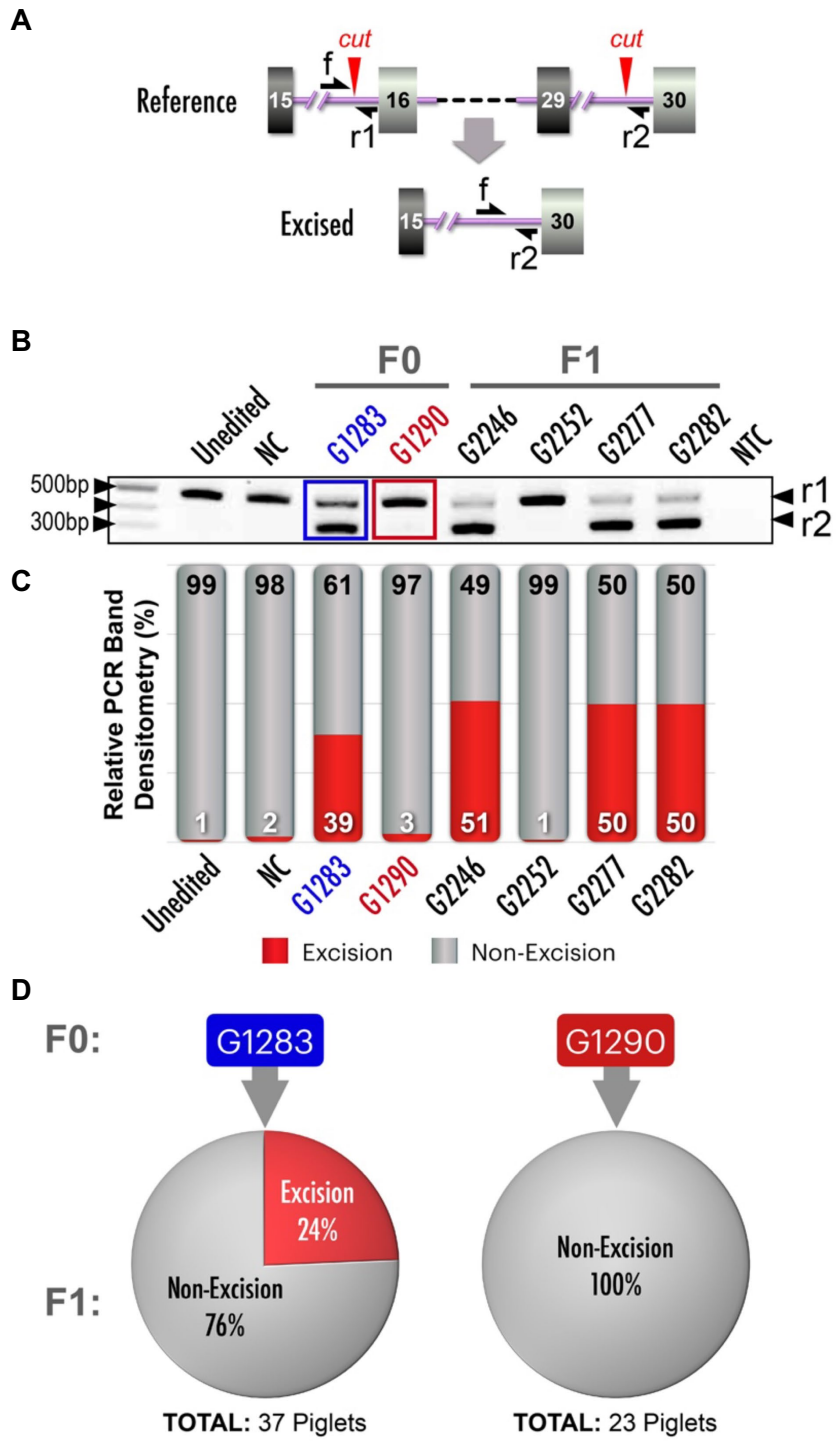
*NF1* 23kb excision and analysis of mosaicism. (**A**) Schematic depicts alterations between *NF1* exons 15/16 and 29/30, resulting in a 23kb excision. Red arrowheads: Cas9 cut sites, single-headed black arrows (f, r1, r2): primer annealing sites. (**B**) Competitive PCR for excision and non-excision alleles yields amplicon products of two different sizes, indicated by black arrowheads on right (r1 and r2). Included are an unedited control, a negative control (NC; a founder (G401) from another unrelated NF1 model, two 23kb excision founders (F0; blue and red labels), F1 offsprings (non-mosaic controls) and no template control (NTC). Black arrowhead (on left): 500bp, 400bp and 100bp markers on DNA ladder (NEB, N0551S). (**C**) Quantification of the pixel density of PCR products in (B) and additional bands not depicted in panel B (see Supplementary Figure 1 for complete gel electrophoresis image), with bars aligned with corresponding PCR gel wells to identify the most effective breeders carrying the 23kb excision. (**D**) Proportionality of F1 pigs from two specific founders in (**B**) and (**C**) carrying the excision allele compared to those not carrying the excision allele. Thirty-nine percent of F1 pigs produced by founder pig G1283 carried an excision allele, while none of the F1 pigs produced by founder pig G1290 did so. There was a significant association between excision allele abundance (pixel density) and germline transmission rate (logistic regression, *p*<0.05).

We next sought to introduce a pathogenic NF1 structural variant, a 23kb excision that included orthologs of human *NF1* exons 16–29 (dbVar: nsv532110; ClinVar RCV000053428.4; Kaminsky et al., 2011). We designed two target sites that flanked the region to be excised (Figure 4A). When tail biopsies from resultant piglets from a single round of embryo microinjections were analyzed, PCR amplification across the intended excision breakpoints revealed two founders carrying the excision, and Sanger sequencing revealed identical excisions in both animals (data not shown). After identifying founders carrying the allele of interest, we sought to strategically identify the most effective breeder to efficiently yield the F1 generation. Similar to the *NF1* Early Truncation (E19^*^) model described above, we presumed mosaicism was present in these animals. To estimate the frequency of the 23kb excision, we designed a simple competitive, endpoint PCR which amplifies the excision allele and the non-excision allele in a single reaction (Pekhletski and Hampson, 1996; Harayama and Riezman, 2017). This assay used a common forward primer and two reverse primers (one against the excision allele and one against the non-excision allele; Figure 4A). The relative abundance of each amplicon at the end point can be interpreted as an approximation of the relative abundance of each allele in the initial sample. While a similar assay could be performed using a relative quantitative PCR strategy (real-time PCR or dPCR), such a strategy requires significantly higher handling time and costs for synthesizing custom probes. While other applications of competitive PCR are commonly used as an alternative qPCR strategy (Gilliland et al., 1990; Edris et al., 1994), our unique application of the method is designed to detect edited *vs*. unedited alleles in a mosaic or pooled cell population.

Gel electrophoresis of competitive PCR reactions revealed differences in abundance of the excision allele from founders G1283 and G1290 (Figure 4B). True heterozygote F1 controls (G2246, G2277, G2282) revealed the expected banding pattern for non-mosaic samples. By semi-quantitating our competitive PCR products, we confirmed that one founder carried the 23kb excision allele at a higher abundance than the other founder (Figure 4C). We predicted this animal would be a more efficient transmitter of the 23kb excision allele. As expected, the founder with a greater abundance of the desired 23kb excision allele measured with competitive PCR gave a higher rate of germline transmission (Figure 4D, logistic regression, *p*<0.05).

### *NF1* a31 Excision (Splice Variant) Model

NF1 gene exhibits notable RNA splicing diversity (Anastasaki et al., 2017; Bergoug et al., 2020) and ∼30% of pathogenic variants cause splicing alterations affecting mRNA processing (Skuse and Cappione, 1997; Messiaen et al., 2000; Wimmer et al., 2007; Valero et al., 2011; Alkindy et al., 2012; Van Minkelen et al., 2014; Evans et al., 2016). We focused on modeling a mutation that would affect the inclusion of alternatively spliced exon 31 (“a31”) [commonly referred to as exon 23a, based on previous nomenclature (Anastasaki et al., 2017)], which is implicated as being important in modulating Ras/ERK signaling as well as learning and memory (Andersen et al., 1993; Costa et al., 2001; Barron and Lou, 2012; Nguyen et al., 2017). A study in a cohort of genetically and clinically characterized NF1 patients stratified according to the severity of the phenotype indicates that increased exclusion of exon a31 is associated with increased severity of phenotype and cognitive impairment (Assunto et al., 2019).

**Figure 5:**
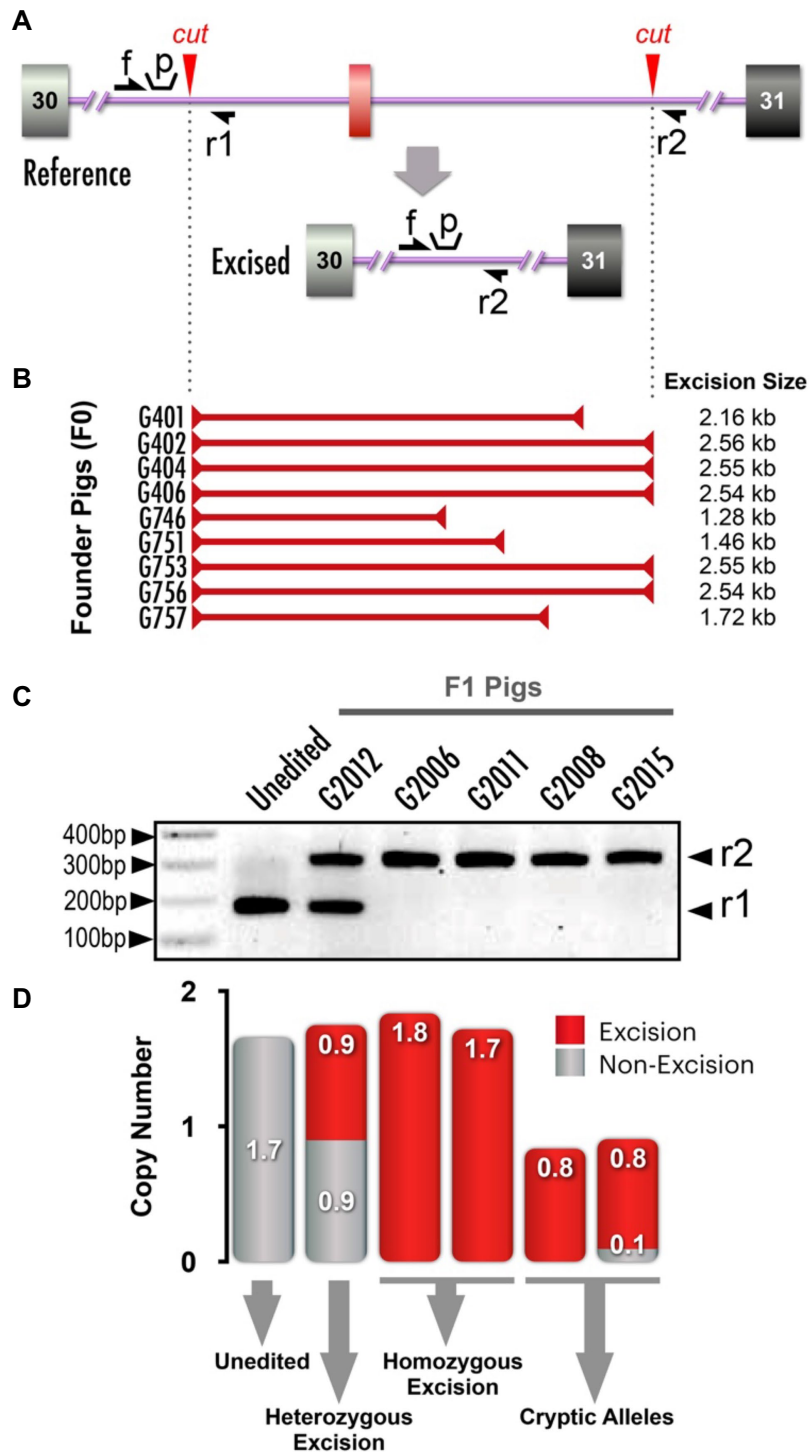
*NF1* splicing variants (*NF1* a31 excision) and copy number variance (CNV) analysis. (**A**) Schematic depicts alterations made within flanking regions of *NF1* exon a31. Red arrowheads: Cas9 cut sites, single-headed black arrows (f, r1, r2): primer annealing sites for competitive and dPCR (distinct primer sets, see Supplementary Table 2 for primer sequences), “p” indicates dPCR probe annealing site. (**B**) PCR analysis identified 10 out of 35 founder pigs as carrying the a31 excision. The breakpoints of each excision allele are mapped to scale, depicting the diversity of recovered excision alleles (only two were identical). Sanger sequencing identified excisions ranging from 1.28kb to the expected 2.56kb. (**C**) PCR amplicon products from F1 pigs using primers indicated in (**A**). See Supplementary Figure 2 for complete gel electrophoresis image. F1 pigs from founders with identical excision alleles revealed the presence of excision and non-excision alleles. Amplification of excision alleles and non-excision alleles yielded products of different sizes, indicated with black arrowheads on right (r1 and r2). Unedited and heterozygote (non-cryptic) are shown, along with four pigs in which only the excision allele was detected (i.e., a non-excision allele was not detected). (**D**) CNV quantification determined separately for excision and non-excision dPCR (red and grey, respectively) and plotted together. Note that for some F1 pigs only a single copy was detected in both PCRs, indicating that these animals carry cryptic mutations that cannot be readily detected.

We designed a 2.5kb excision flanking exon a31 (Figure 5A). PCR analysis of genomic DNA from tail biopsies of resultant piglets from four rounds of microinjection identified 10 of 35 pigs to be carrying an excision of the area flanking exon a31. The excision breakpoints varied (Figure 5B) and were confirmed with Sanger sequencing (data not shown).

As we wanted to generate homozygous F1 pigs rapidly for a study, we crossbred two founders carrying exon a31 excisions. By Mendelian genetics, we expected F1 pigs to carry a pair of alleles traceable to each parent. When F1 pigs appear to carry a pair of the same alleles (i.e., a single PCR band is detected), the classic interpretation would be that these F1 pigs are homozygous, as they inherited the same allele from both parents. However, in some instances, we observed that the single allele detected in F1 pigs was not detected in both parents. Therefore, we suspected a second allele was present, but was not detectable by PCR (a “cryptic allele”). Presumably, these cryptic alleles arose from large rearrangements or structural variants generated during the process of double-strand break (DSB) repair.

While CRISPR provides an efficient way to edit the genome at targeted locations, several studies in cell lines and animal models have reported the unexpected generation of structural variants that can be very difficult to detect through standard approaches such as PCR analysis (Shin et al., 2017; Kosicki et al., 2018; Rezza et al., 2019; Korablev et al., 2020). Backcrossing of founders is a common practice in mouse model creation to avoid transmission of unexpected variants, including undetectable alleles at the target locus (e.g., structural variants). However, this approach is neither practical (can add years to the process) nor cost-effective in porcine model creation.

To definitively assess this, we used a dPCR-based CNV assay to count the number of alleles detectable through PCR amplification. In normal circumstances, we would expect our primer designs to capture all possible alleles, thus providing a CNV quantification of 2. In the circumstance where an F1 pig received an expected allele from one parent and an unidentified cryptic allele from the other parent, our PCR primers would not be able to detect the cryptic allele and thus would yield a CNV quantification of 1.

We encountered a representative situation that highlights the essentiality of CNV analysis. When we bred two founders carrying identical exon a31 excisions, we recovered F1 pigs that were seemingly heterozygous or homozygous for the excision allele (Figure 5C). Next, we designed two dPCR assays to separately measure the CNV quantifications of the excision and non-excision alleles (Figure 5A and Supplementary Table 2 for primer design). We expected the sum of excision and non-excision CNV quantifications to be 2, as this would indicate we are able to amplify both alleles present in the pig. However, we found several animals that had total CNV quantifications of 1 (Figure 5D). In the example shown, we were only able to amplify a single excision allele, but the animal’s second allele could not be amplified by either CNV assays. Without this CNV analysis, these F1 pigs would have been incorrectly presumed to be homozygous for the excision.

When we applied CNV analysis to the founder pigs of our *NF1* a31 excision model herd, we observed a range of CNV values from 1 to 2 (data not shown), indicating that cryptic alleles are not rare. Furthermore, these alleles were transmitted to the F1 pigs. Further examination of founder pigs of our other models revealed this phenomenon may be universal when using CRISPR in pigs. Using this dataset, we were able to distinguish pigs that were *bona fide* homozygotes from pigs carrying an excision allele and also a cryptic allele. This method reliably allows us to generate validated homozygous F1 pigs after only a single round of breeding.

### *TP53* Loss of Function (LOF) Model

**Figure 6:**
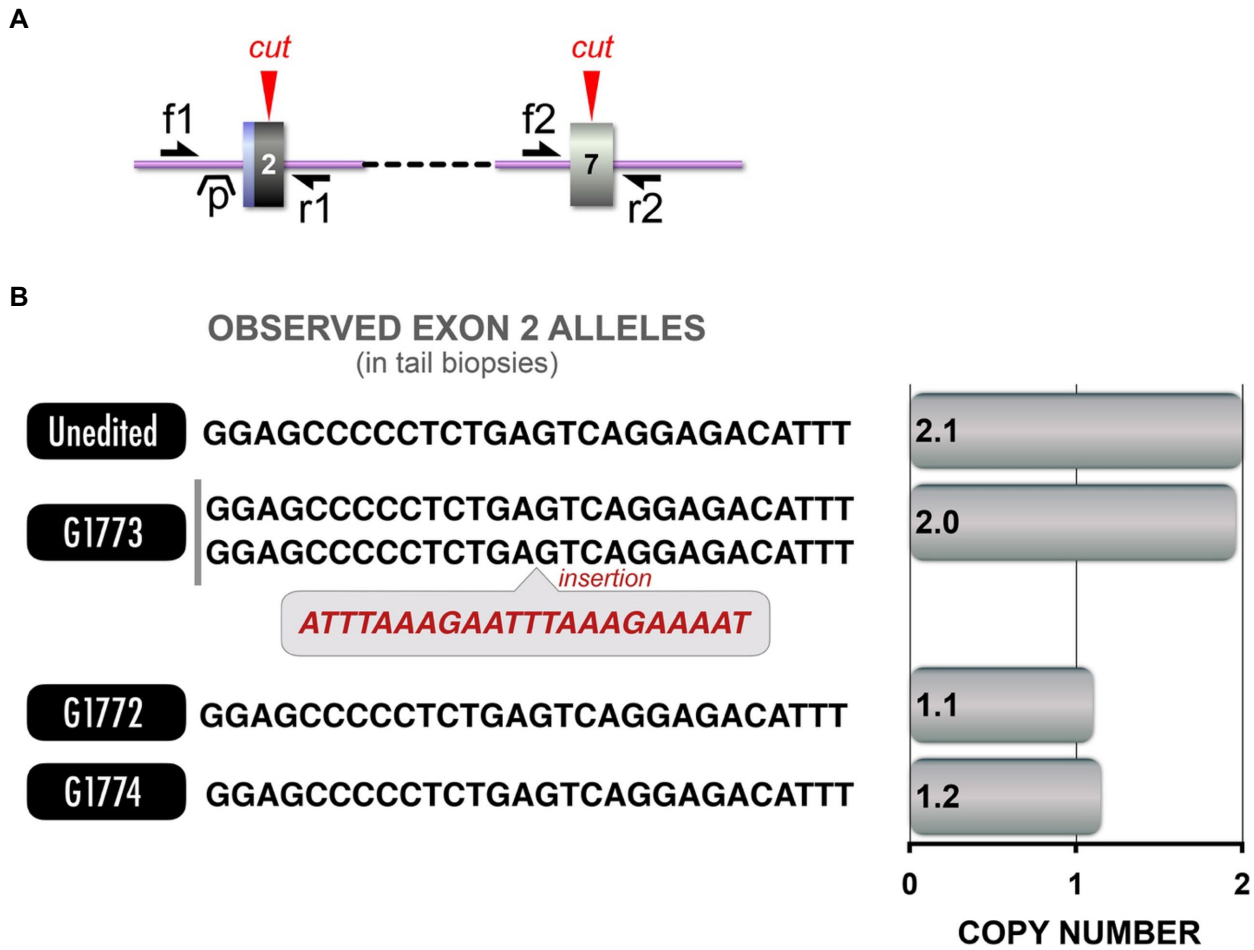
*TP53* LOF model and CNV analysis. (**A**) Schematic depicts Cas9 target sites in exon 2 and exon 7 of *TP53*. Red arrowheads: Cas9 cut sites, single-headed black arrows (f1, f2, r1, r2): primer annealing sites for competitive and dPCR (distinct primer sets, see Supplementary Table 2 for primer sequences), “p” indicates dPCR probe annealing site. (**B**) Left: Allele sequences from each pig are provided, along with the CNV analysis corresponding to those sequences. Right: CNV analysis revealed that while some pigs carried the excision, others carried alleles that could not be amplified.

The inactivation of *TP53* tumor suppressor gene in tumors of NF1 patients has been implicated in the progression of these tumors into malignancy (Cichowski and Jacks, 2001), including malignant astrocytoma (Zhu et al., 2005), malignant neurofibrosarcomas (Menon et al., 1990), and MPNST (Halling et al., 1996). To further our study of tumorigenesis observed in our NF1 pig models and the cooperative role that *TP53* inactivation plays, we sought to generate *TP53* mutant pigs that could then be crossbred with any of the NF1 models to provide the desired combination. We designed gRNAs targeting two *TP53* sites (Figure 6A). An upstream exon 2 target site was selected to provide an early truncation and a strong loss-of-function allele, while a downstream exon 7 site which mimics a hot spot for frameshifting truncations. Gain of function truncating mutations near the latter site promotes tumorigenesis (Shirole et al., 2016). TAm-Seq to characterize the alleles found in the resultant piglets from two rounds of microinjections observed 10 pigs (out of 29 total pigs) carried indels at exon 2, while none carried indels at exon 7. Two others were found to carry excisions between exon 2 and exon 7, identified through PCR.

Similar to the *NF1* a31 Excision model, we crossbred the two *TP53* founder pigs to rapidly generate *TP53* LOF pigs. As before, we observed (by sequencing) F1 pigs that appeared to be unexpectedly homozygous for an allele present in only one parent, suggesting cryptic alleles were segregating in F1 pigs. Indeed, CNV analysis of the F1 pigs (Figure 6B) confirmed the presence of a cryptic *TP53* allele which we were unable to amplify with PCR. These results shows that the phenomenon of cryptic alleles is not unique to CRISPR editing of any specific gene (e.g., *NF1*).

At the stage when we designed the target sites for our *TP53* LOF model creation, tools for identifying swine Cas9 target sites were limited. Retrospectively, we discovered that MIT’s CRISPR design tool masked some genomic regions. When CRISPOR became available (Concordet and Haeussler, 2018) and we queried our target sites, we found that a *TP53* pseudogene was also targeted by the same target sequence used in our previous experiments. We identified a retroposition of the *TP53* gene (ENSSSCG00000014618), which was also modified in at least two founder animals. We were able to rapidly and efficiently screen over a hundred samples (all founders and all subsequent progeny) for unintended edits at this locus because of the highly parallel, cost-efficient nature of TAm-Seq.

### Conclusion

As genome-edited porcine model creation and utilization becomes increasingly pervasive, it is incumbent upon the community to ensure best practices for reliable genotyping, including strategic determination of optimal pipelines and quality assurance benchmarks to introduce these edits. Our work here demonstrates key methods that efficiently provide solutions where previous porcine genome-editing work have left off.

**Figure 7:**
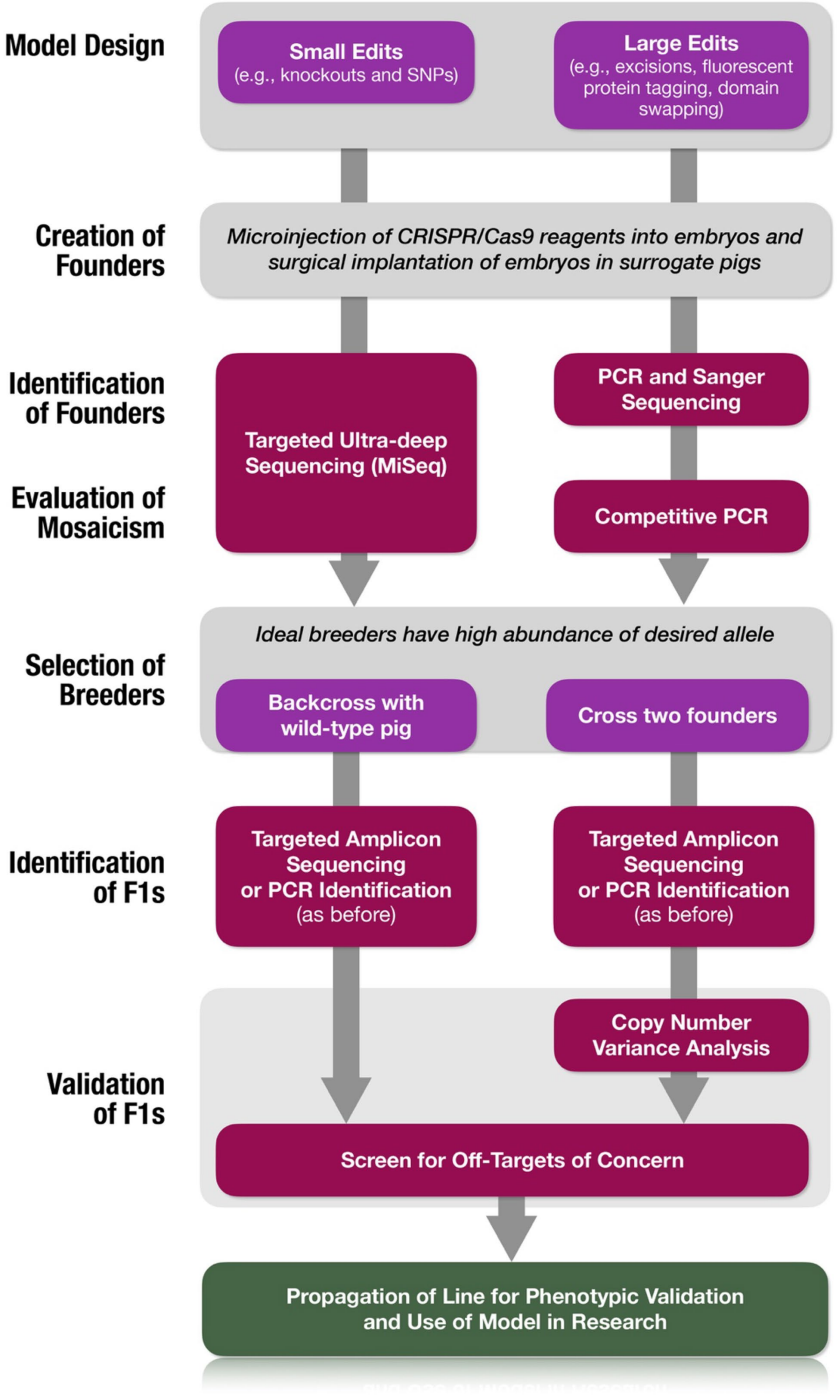
Simplified workflow for creation of validated swine models. Decision trees are provided for the type of edit being introduced as well as the strategy for the utilization of founders for the expansion of the line.

Simple methods to assess mosaicism in founders and identify ideal breeders are presented. Two simple strategies for small edits (TAm-Seq) or larger edits such as excisions, insertions or domain swapping (competitive PCR) are offered (Figure 7). While further considerations can be made to reduce mosaicism, we are not able to address those here. Such strategies include utilizing Cas9 protein rather than mRNA or plasmid (Hashimoto et al., 2016; Mehravar et al., 2019; Hennig et al., 2020), careful titration of Cas9 concentration, and optimization of edit timing (Lamas-Toranzo et al., 2019; Tanihara et al., 2019).

We also provide a framework (Figure 7) to identify *bona fide* biallelic homozygous founders and to confidently generate homozygous mutations when *bona fide* biallelic founders are not available. Our method makes no presumptions about the nature of underlying, unidentified cryptic mutations, but simply identifies these alleles in the sample by omission. Thus far, these cryptic alleles have evaded our attempts at molecular characterization. More comprehensive—and expensive—solutions include the use of whole genome sequencing. While costs of short-read Illumina whole genome sequencing have steadily declined, this analysis is not sufficient to confidently assert the absence of structural variants that occur in response to Cas9-mediated DSB formation. Long-read whole genome sequencing technologies (i.e., Pacific Biosciences and Oxford Nanopore Technologies) would more likely provide identification of these alleles but are not scalable and/or are considerably more expensive. Additional reasonably scalable approaches include nanopore-based Cas9-mediated selective long-read sequencing (Gilpatrick et al., 2020) and unidirectional targeted sequencing (Giannoukos et al., 2018), and we hope these methods will lead to the successful characterization of these alleles. We aimed to present a streamlined, laboratory-standard, cost-effective strategy for generating biomedical genome-edited models by embryo microinjection.

This report also provides a toolkit for biomedical porcine models of NF1. We have generated two lines of porcine models with patient-specific mutations and one with alternative splicing defect. Several combinations of these lines harboring *TP53* LOF have also been produced. These lines can be used to explore NF1-related tumor formation and non-tumor phenotypes and to understand the role of complex *NF1* splicing diversity. In addition to the more fundamental phenotypes such as café au lait spots and neurofibromas, our NF1 models show considerable diversity and complexity of phenotypes similar to those often observed in human patients. An extensive characterization of pathology and histopathology and observed genotype–phenotype associations are outside the scope of the current manuscript and will be published elsewhere.

## Data Availability Statement

The raw data supporting the conclusions of this article will be made available by the authors, without undue reservation.

## Ethics Statement

The animal study was reviewed and approved by University of Wisconsin–Madison Institutional Animal Care and Use Committee.

## Author Contributions

CR, BL, JM, DTS, KK, JR, MM, MA, CK, and DS contributed to conception or design. CR, DM, BL, JM, DTS, KK, JR, MA, CK, and DS contributed to data acquisition, analysis, and interpretation. CR, DM, BL, JM, DTS, and DS drafted the manuscript, and CR, BL, JM, DTS, KK, JR, MM, MA, CK, and DS critically revised the manuscript. All authors gave final approval and agreed to be accountable for all aspects of work in ensuring that questions relating to the accuracy or integrity of any part of the work are appropriately investigated and resolved. All authors contributed to the article and approved the submitted version.

## Funding

This work was supported by funding from the Biomedical & Genomic Research Group Discretionary Fund (University of Wisconsin-Madison), Neurofibromatosis Network, NF North Central, NF Team, and Links for Lauren. This work was also supported by the Office of the Assistant Secretary of Defense for Health Affairs and the Defense Health Agency J9, Research and Development Directorate, through the Neurofibromatosis Research Program (NFRP) under Award No. W81XWH-18-1-0633. Opinions, interpretations, conclusions, and recommendations are those of the author and are not necessarily endorsed by the Department of Defense. Additional support for RRID:SCR_017759 was provided by the University of Wisconsin Carbone Cancer Center (NIH/NCI funding: 5P30CA014520-40)

## Conflict of Interest

The authors declare that the research was conducted in the absence of any commercial or financial relationships that could be construed as a potential conflict of interest.

## Publisher’s Note

All claims expressed in this article are solely those of the authors and do not necessarily represent those of their affiliated organizations, or those of the publisher, the editors and the reviewers. Any product that may be evaluated in this article, or claim that may be made by its manufacturer, is not guaranteed or endorsed by the publisher.

## Footnotes

^1^ https://zlab.bio/guide-design-resources
