# Supplementary material for: Assessment of Mosaicism and Detection of Cryptic Alleles in CRISPR/Cas9-Engineered Neurofibromatosis Type 1 and TP53 Mutant Porcine Models Reveals Overlooked Challenges in Precision Modeling of Human Diseases
Source: Front Genet. 2021 Sep 23;12:721045. doi: 10.3389/fgene.2021.721045 (PMC8495252; doi:10.3389/fgene.2021.721045)

Supplementary Figure 1

Complete gel electrophoresis image of *NF1* 23kb excision PCR in Figure 4C. Note the presence of doublet bands in all samples carrying the NF1 23kb excision. For densitometric analysis, these bands were summed with the r1 excision band labeled in Figure 4C.

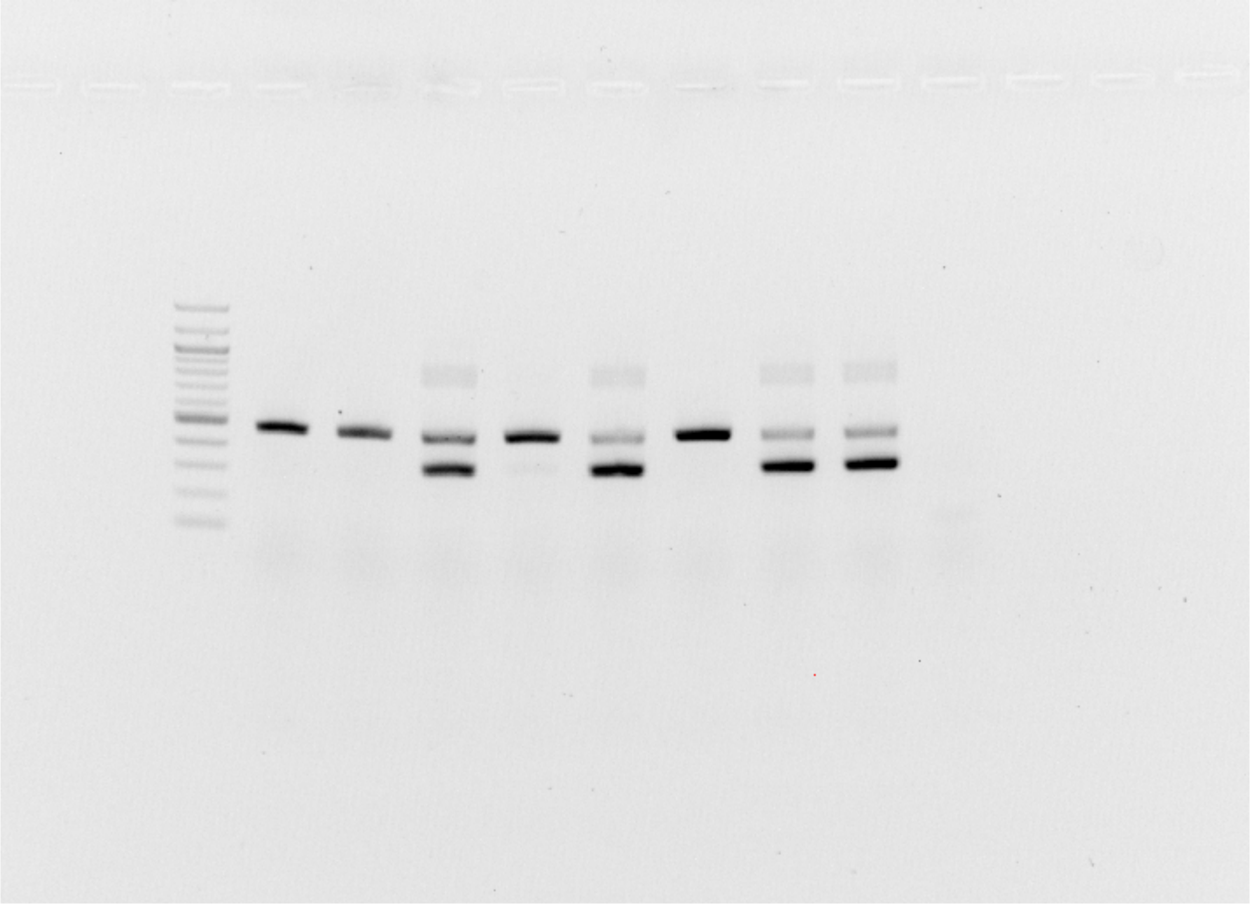

Supplement: Supplementary file 2 [file Image_1.PDF]
