# Supplementary figures and images for: Assessment of Mosaicism and Detection of Cryptic Alleles in CRISPR/Cas9-Engineered Neurofibromatosis Type 1 and TP53 Mutant Porcine Models Reveals Overlooked Challenges in Precision Modeling of Human Diseases
Source: Front Genet. 2021 Sep 23;12:721045. doi: 10.3389/fgene.2021.721045 (PMC8495252; doi:10.3389/fgene.2021.721045)

## Supplementary Figure 2

Complete gel electrophoresis image of *NF1* a31 excision in Figure 5C.

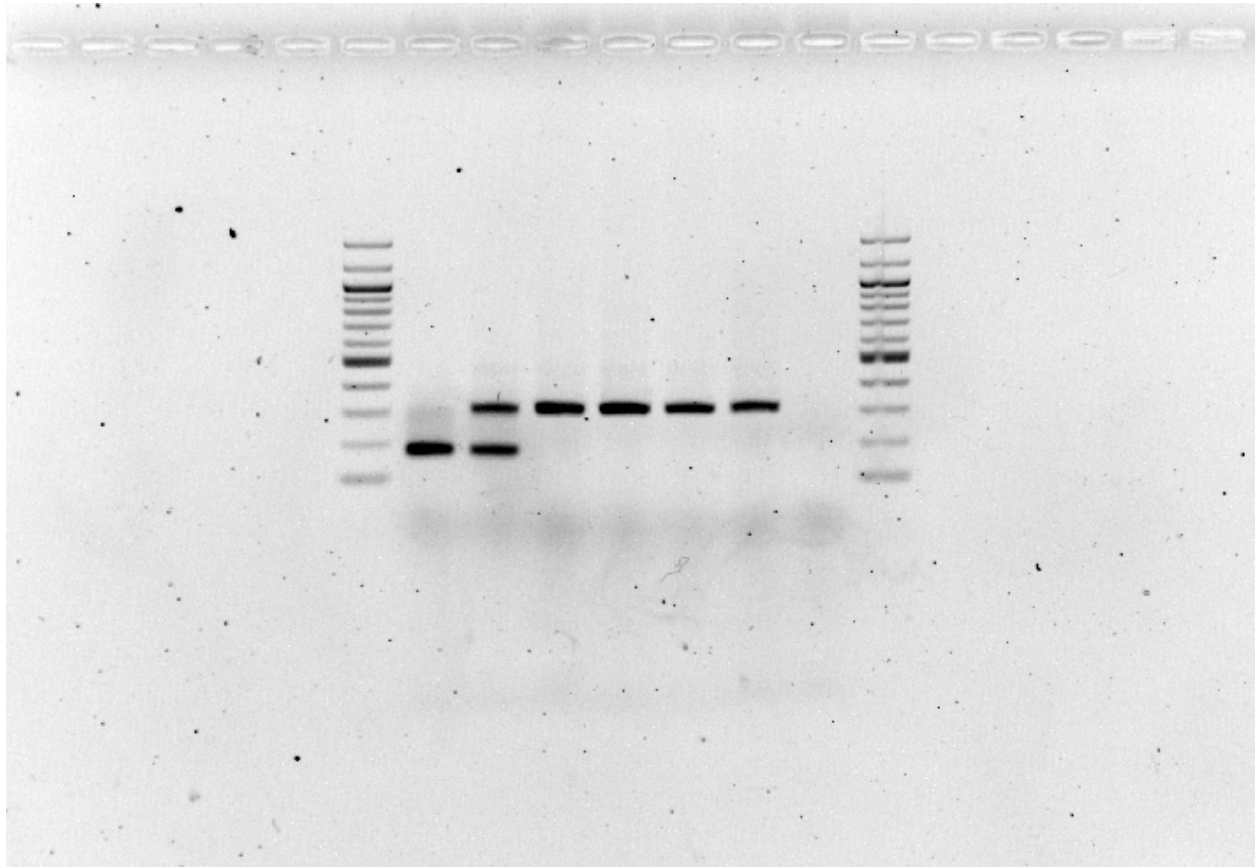

Supplement: Supplementary file 3 [file Image_2.pdf]
